# Air pollution as a contributor to the inflammatory activity of multiple sclerosis

**DOI:** 10.1186/s12974-020-01977-0

**Published:** 2020-11-06

**Authors:** Andrea Cortese, Luca Lova, Patrizia Comoli, Elisabetta Volpe, Silvia Villa, Giulia Mallucci, Sabrina La Salvia, Alfredo Romani, Diego Franciotta, Valentina Bollati, Sabrina Basso, Ilaria Guido, Giuseppe Quartuccio, Luca Battistini, Cristina Cereda, Roberto Bergamaschi

**Affiliations:** 1 Department of Brain and Behavioural Sciences, University of Pavia, Pavia, Italy; 2 Department of Neuromuscular Diseases, UCL Queen Square Institute of Neurology, Queen Square, London, UK; 3 Biosciences S.p.A., Milan, Italy; 4 IRCCS Policlinico S. Matteo Foundation, Pavia, Italy; 5 IRCCS Santa Lucia Foundation, Rome, Italy; 6 IRCCS Mondino Foundation, Pavia, Italy; 7 University of Milan, Milan, Italy

**Keywords:** Air pollution, Particulate matter, Multiple sclerosis, Th 17 lymphocytes, Adhesion molecules

## Abstract

**Abstract:**

**Objective:**

Air pollution has been recently identified as a risk factor for multiple sclerosis. Aim of this study was to investigate the immunological mechanism underlying the clinical association between air pollution, namely exposure to particulate matter 10 (PM10), and inflammatory activity of multiple sclerosis (MS)

**Methods:**

Daily recording of PM10 was obtained by monitors depending on the residence of subjects. Expression of molecules involved in activation, adhesion, and migration of T lymphocytes were tested by flow cytometry in 57 MS patients and 19 healthy controls. We next assessed in vitro the effect of PM10 on expression of C-C chemokine receptors 6 (CCR6) by peripheral blood mononuclear cells (PBMCs), on cytokine production by monocyte-derived dendritic cells (mdDC), and on T cell polarization in PBMC/mdDC mixed cultures.

**Results:**

We identified a significant correlation between mean PM10 levels and expression of CCR6 CD4+ T circulating cells in MS patients. This was paralleled by the observation in vitro of a higher level of CCR6 expression on PBMC following treatment with increased doses of particulate matter. Moreover, in mdDC cultures, particulate matter induced the secretion by mdDC of Th17 polarizing IL1 beta, IL6, and IL23 and, in mdDC/PBMC mixed cultures, enhanced generation of IL17-producing T cells.

**Conclusions:**

Ex vivo and in vitro studies support the pro-inflammatory role of PM in MS, by upregulating expression of CCR6 on circulating CD4+ T cells and inducing in innate immune cells the production of Th17 polarizing cytokines. Therefore, we speculate that in MS respiratory exposure to PM10 may induce the production in the lung of autoreactive Th17 lymphocytes and boost their migratory properties through the blood-brain barrier.

## Introduction

Multiple sclerosis (MS) is an inflammatory demyelinating disorder of the central nervous system (CNS) which is characterized in its early phase by periods of exacerbation and remission along with progressive neurologic disability. During MS relapses, inflammatory cells migrate through the blood-brain barrier (BBB) inside the CNS, where autoreactive T cells are reactivated, leading to local inflammatory demyelination, disruption of the BBB, and subsequent enhanced extravasation of leukocyte [1, 2].

MS relapse occurrence varies during the year, and different studies reported an increased rela pse rate in either autumn/winter or spring/summer [3]. Infections, low vitamin D, and low melatonin levels have been associated to increase relapse occurrence [4, 5]. Moreover, dietary habits, by modifying the gut microbiota [6, 7], stress, and smoking have also been linked to increase incidence and MS exacerbation [8]. However, none of these factors can fully explain the variability of MS course, thus suggesting that additional factors may play a role.

In a recent study, we have established a significant association between air-borne particulate matter levels and inflammatory activity of the disease, as expressed by the presence of gadolinium-enhancing lesions on brain MRI in MS patients [9]. Other groups have also observed an association between ambient levels of air pollution and MS prevalence [10], as well as clinical activity of the disease [11–14].

Altogether, these observations support a role for air pollution as a novel risk factor for MS, favoring its inflammatory exacerbations. However, the mechanisms underlying this clinical observation remain unknown.

Therefore, here, we investigated by the effect of the exposition to particulate matter 10 (PM10) on inflammatory markers on circulating CD4 and CD8 lymphocytes in MS patients and controls. As an association between PM10 and the expression of the migration marker CCR6 was identified, we performed in vitro studies in order to further confirm the effect of PM10 on its expression, as well as on cytokine production and T cell polarization.

## Methods

### Subjects

Venous blood samples were obtained between December 2013 and April 2015 from 57 prospectively enrolled relapsing remitting (RR) MS patients with clinically and radiologically stable disease and 19 healthy controls, who were recruited among people having the same residence of enrolled MS patients. Mean age of patients was 43 ± 10 years, 24 (42%) were males, 15 (26%) were smoker, 31 (54%) were on disease modifying treatment. Mean EDSS was 1.4 ± 1.5 (range 0–6), and mean disease duration was 7.8 ± 9 years. Mean age of controls was 46 ± 12 years; 10 (53%) were males; 5 (26%) were smoker.

The Ethics Committee of San Raffaele Hospital, Milan, Italy, approved study protocol, and all subjects signed informed consent form.

### Air pollution measurement

Ambient concentrations of PM10, which is physically defined by the mass median aerodynamic diameter < 10 μm of the pollutant particles, were obtained from the regional air-quality monitoring network (ARPA, Regional Environmental Protection Agency). PM10 concentrations were obtained from the seven stations located in the Pavia province, as all the study participants were resident in that area.

The addresses of monitoring stations and study subjects were geocoded in order to assign to each subject the daily PM10 concentration from the nearest monitor to home address. None of the cases enrolled reported the use of home air filters. For patients who worked far from their residence, mean daily PM10 levels at residence and work place were averaged. PM10 exposure was considered from the date of blood draw to the 15 days before. The 15 days’ time period was chosen based on our previous work showing an association between the presence of gadolinium-enhancing lesion on brain MRI and the level, which was higher at 15 days prior to the MRI scan [9]. Therefore, mean PM10 level 15 days prior to blood draw was also used in the models of this study.

### Flow cytometry

Blood samples were freshly analyzed by lyse-and-wash whole blood staining procedure. Surface staining was performed on ice for 20 min, and the cells were then analyzed on a two laser, six color FACSCanto flow cytometer. Multiplexed dilutions of monoclonal antibodies (mAbs) were used to characterize lymphocyte populations. The following antibodies from BD Biosciences were used: CD69-FITC, HLA-DR-FITC, CD49d-PE, CD11a-PE-Cy7, CD4APC-H7, CD8 APC-H7, CCR6-PerCP-Cy5.5, CD183 PE-Cy 7, CD44- PerCP-Cy 5.5.

### PBMC and mixed mdDC/PBMC cultures

PBMCs were separated by Ficoll-Hypaque centrifugation (Amersham Biosciences) from buffy coats of healthy blood donor volunteers and were cultured according to standard procedures in presence of different doses of particulate matter (1648a, urban particulate matter, National Institute of Reference Material, USA; final concentration used after optimisation 5–20 μg/ml for PBMC and 10–40 μg/ml for mdDC and mdDC/PBMC co-cultures). mdDC were generated from peripheral blood monocytes as described previously [15]. For mixed cultures, PBMC were cocultured with unmanipulated mdDC, or with mdDC pulsed with PM10 (PBMC:mdDC ratio of 100:1), for 8 days in RPMI medium supplemented with 10% fetal calf serum (Hyclone). Cytokine secretion profile was evaluated on culture supernatant by ELISA and on cultured cells by ELISPOT assay.

### ELISPOT and ELISA assays

ELISPOT assays were performed according to a previously described method [15]. Briefly, 96-well multiscreen filter plates (MAIPS 4510, Millipore, Bedford, MA) were coated with 100 μl of primary antibody (IL17; Mabtech, Nacka, Sweden) at 2.5 μg/ml, and incubated overnight at 4 °C. Cultured cells were plated at 1 × 105 cells/well in triplicate. After incubation for 24 h at 37 °C, 100 μl of biotinylated secondary antibody (Mabtech, 0.5 μg/ml) was added, and plates were then processed according to standard procedure. Cytokine-producing spots were counted using an ELISPOT reader (Bioline, Torino, Italy). The number of spots per well was calculated after subtracting the assay background, quantitated as an average of 24 wells containing only sterile complete medium, and specific background, quantitated as the sum of cytokine spots associated with responders alone.

Cytokine levels in the supernatant of DCs or DC-PBMC cocultures were measured using monoclonal antibody pairs (IL1 beta and IL17: Mabtech; IL6: Endogen Tema, Castenaso, Italia; IL23: U-Cytech Biosciences, Utrecht, The Netherlands). Plates were coated with purified antibodies at the appropriate concentrations. Standard curves were prepared with recombinant human cytokines. Biotin-labeled antibodies were added and HRP-conjugated streptavidine was used to develop the reactions. Plates were read at 450 nm (Titertek Plus MS 212 M). Results were reported as picograms per milliliter.

### Statistical analyses

The sample was described by means of mean and standard deviation for continuous variables and proportions for categorical ones. Spearman’s coefficient was used to test the correlation between mean fluorescence intensity for the molecules tested and PM10 levels. Association between correlated molecules and PM10 was further tested in a multivariate linear regression model including age, gender, treatment with disease-modifier drugs (yes/no), and smoker status. Continuous variables compared different PM exposure conditions tested in vitro using non-parametric or parametric two-tailed paired or unpaired Student’s *t* test. Statistical significance was taken at the < 0.05 level. All analyses were conducted using STATA/SE for Windows, version 12.1 (StataCorp, College Station, TX, USA). Graphs were generated using the GraphPad Prism v7 Software (San Diego, CA, USA).

## Results

### In MS patients, PM10 boosts the expression of CCR6 on circulating lymphocytes

**Fig. 1:**
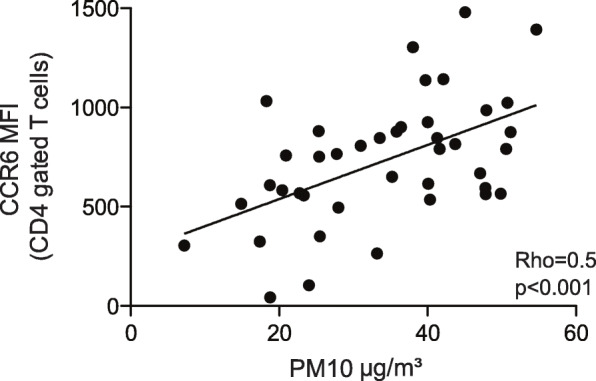
Scatterplot of expression of CCR6 on CD4+ T cells in MS patients. CCR6: chemokine receptor 6; MFI: mean fluorescence intensity

In MS patients, but not in controls, we observed a significant correlation between mean PM10 level in the previous 15 days and expression on CD4+ T cells of CCR6, a chemokine receptor characteristic of Th17 cells (Fig. 1 and Tables 1 and 2).

**Table 1:** Mean and standard error of expression level, here indicated as mean fluorescence intensity (MFI), of the tested molecules on CD4+ and CD8+ in MS patients and healthy controls

	CD4 T cells		CD8 T cells	
	MS patients (*n* = 57)	Healthy controls (*n* = 19)	MS patients (*n* = 57)	Healthy controls (*n* = 19)
**Activation markers**				
CD69	224 ± 7 (*n* = 31)	205 ± 8 (*n* = 9)	280 ± 10 (*n* = 30)	285 ± 13 (*n* = 9)
HLA DR3	1008 ± 93 (*n* = 21)	1376 ± 392 (*n* = 8)	1420 ± 139	1406 ± 380 (*n* = 8)
CD38	586 ± 88 (*n* = 21)	476 ± 94 (*n* = 8)	735 ± 92	519 ± 110 (*n* = 8)
**Adhesion and migration markers**				
CD44	**31,582 ± 1321**	**23,702** ± **3122**	**26,553 ± 1229**	**17,792** ± **2288**
VLA4	2300 ± 85	2518 ± 185	3102 ± 108	3214 ± 161
LFA1	6073 ± 242	7140 ± 561	10594 ± 373	12436 ± 1041
CCR6	739 ± 42 (*n* = 55)	808 ± 72 (*n* = 18)	478 ± 28 (*n* = 55)	575 ± 52 (*n* = 18)
CXCR3	667 ± 40	861 ± 111	815 ± 59	736 ± 97

**Table 2:** Correlation between average PM10 levels 15 days before enrolment and expression level of the all tested molecules on CD4+ and CD8+

Spearman’s Rho between mean of PM10 15 days before enrolment and:	Healthy Controls (***n*** = 19)		MS patients (***n*** = 57)	
	CD4 T cells	CD8 T cells	CD4 T cells	CD8 T cells
**Activation markers**				
CD69	− 0.2, *p* = 0.58	0.22, *p* = 0.55	0.08, *p* = 0.66	− 0.07, *p* = 0.70
HLA DR3	0.22, *p* = 0.60	0.35, *p* = 0.40	− 0.14, *p* = 0.53	− 0.30, *p* = 0.18
CD38	0.31, *p* = 0.45	0.36, *p* = 0.38	0.004, *p* = 0.99	0.01, *p* = 0.96
**Adhesion and migration markers**				
CD44	0.13, *p* = 0.6	0.45, *p* = 0.06	0.22, *p* = 0.1	0.23, *p* = 0.08
VLA4	− 0.05, *p* = 0.84	0.13, *p* = 0.6	0.17, *p* = 0.21	0.12, *p* = 0.36
LFA1	0.02, *p* = 0.92	0.17, *p* = 0.5	0.26, *p* = 0.05	− 0.11, *p* = 0.44
CCR6	− 0.02, *p* = 0.93	− 0.22, *p* = 0.37	**0.43**, ***p*** **=** **0.001**	0.23, *p* = 0.09
CXCR3	− 0.02, *p* = 0.94	− 0.01, *p* = 0.96	0.10, *p* = 0.44	0.03, *p* = 0.83

Values indicated are Spearman’s Rho coefficient, *p* value

In a linear regression model, the association between exposure to PM10 and the expression of CCR6 (*β* = 7.8, 95% CI = 3.2–12.5, *p* < 0.001) on CD4+ T cells was independent from age, sex, treatment, and smoker status (Table 3). A weak correlation was also observed between PM10 levels and expression of LFA1 on CD4+ T cells, which was not confirmed in an age-, sex-, treatment- and smoker status-adjusted regression model.

**Table 3:** Multivariate analysis showing the association of CCR6 expression on CD4+ T cells and PM10 level

	Coeff. [95% Conf. interval]	***p*** > |***t***|
**Mean PM10 level**	**5.7 [1.8–9.7]**	***p*** **= 0.005**
**Age**	− 4.8 [− 11.5–1.81	*p* = 0.15
**Male Gender**	− 22.4 [− 164.2–119.3]	*p* = 0.75
**Disease modifying therapy**	− 38.3 [− 187.8–111.1]	*p* = 0.6
**Smoker**	− 105.6 [− 263.2–51.9]	*p* = 0.18

Patients were followed up after 3 months, but none of them had a clinical relapse, thus preventing further analysis of the correlation between expression of activation and adhesion molecules and clinical activity of the disease in our study population.

The association between CCR6 expression on CD4+ T cells, marker characteristic of the Th17 lineage, and PM10 may suggest a prominent effect of PM10 exposure on Th17-driven inflammatory response.

### In vitro PM10 induces expression of migratory markers on PBMCs and enhance DC-dependent generation of IL17-producing T cells

**Fig. 2:**
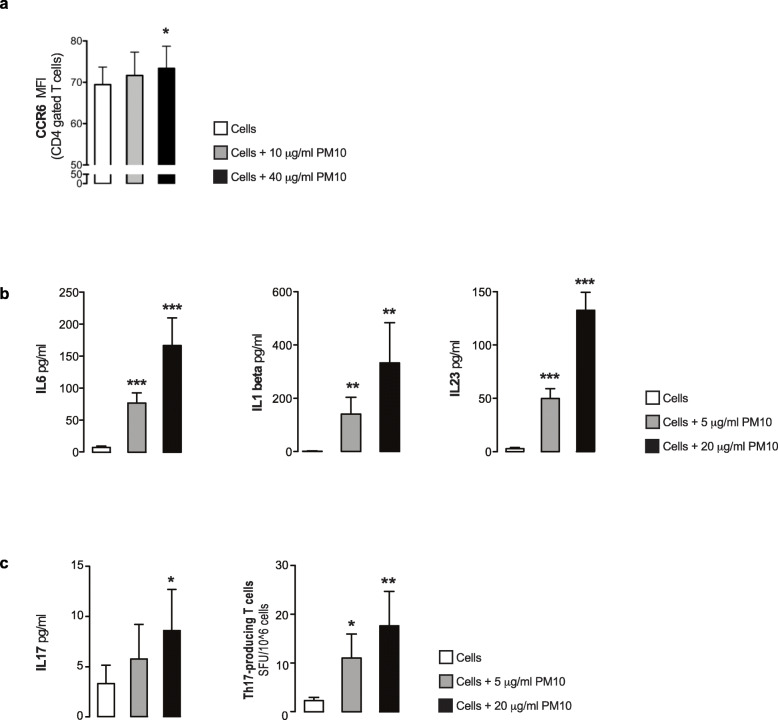
In vitro treatment of PMBCs with PM induces CCR6 expression on CD4+ cells **(a)**. In vitro treatment of mdDCs with increasing doses of PM10 induces secretion of IL-6, IL1 beta, and IL-23 **(b)**. In mixed mdDC-PBMC co-cultures, in vitro treatment of mdDC PM induces release of IL-17and increases the percentage of IL-17 producing T cells **(c)**. *p<0.05, **p<0.001, ***p<0.0001

We next tested in vitro the effect of urban PM on expression of CCR6 as well as on cytokine production and CD4+ T cell polarization. PM10 increased the expression of CCR6 on CD4+ T cells (Fig. 2a). Based on the hypothesis that the effect of PM10 on amplification of CCR6+ Th17 cells may be mediated by innate immunity cells residing in the lung where they may uptake air-born PM, we co-cultured PBMCs with PM-treated mdDC and analyzed IL1 beta, IL6, and IL23 cytokine production by mdDC and assessed in mdDC/PBMCs mixed cultures the effect of PM treatment on Th17 cell polarization. In vitro treatment of mdDC with PM, even at low concentration, induced a massive release of all Th17-polarizing cytokines tested, IL1 beta, IL6, and IL23 (Fig. 2b). As well known, these cytokines are necessary for differentiation of Th17 into fully pathogenic cells [16, 17]. Co-culture of PBMCs with PM-treated mdDC significantly increased the number of IL17-producting T cells on ELISPOT-based assay and increased IL17 cytokine level in cell culture medium (Fig. 2c).

These findings support both presence of a direct effect of PM10 on expression of CCRs on CD4+ T cells and a predominantly indirect effect of PM10 on Th17 polarization, by increasing the production of Th17 polarizing cytokines by dendritic cells.

## Discussion

Incidence of autoimmune disorders has steadily increased in the past century in industrialized countries, thus suggesting that a change in their environment may underlie this epidemiological observation.

Air pollution is increasingly recognized as a major public health concern, and an increased concentration of airborne PM, one of the most studied component of air pollution, has been associated with both increased morbidity and mortality [18].

PM is a complex mixture of constituent chemicals. It is often classified by particle size, although this physical classification may oversimplify the molecular makeup, which may vary from urban to rural areas, including elemental and organic carbons, metals, sulfates, nitrates, and microbial contaminants [19].

Previous studies by us and others found an association between concentration of PM10 in the weeks preceding MS inflammatory activity, demonstrated both by clinical exacerbations [11] and by detection of contrast-enhancing lesions on brain MRI [9].

The findings of the present research suggest that air pollution may lead to MS inflammation by two different mechanisms: (1) PM10 may directly lead to upregulation of CCR6 on circulating lymphocytes, which may facilitate their entry to the CNS; (2) PM10 could increase IL1 beta, IL6, and IL23 production by DC and enhance DC-dependent generation of IL17-producing T cells.

Migration of pathogenic self-reactive T cells to the CNS is an essential step for the development of EAE in immunized mice [20]. Migration of lymphocytes through the BBB is strictly controlled and requires the sequential interaction of AMs on T cells and BBB endothelial cells. In particular, CCR6 is known to mediate early recruitment of Th17 lymphocytes to CNS through its interaction with CCL20 (Chemokine Ligand 20) on choroid plexus and mice lacking CCR6 were shown to be highly resistant to the induction of EAE [21], and increasing evidence support a crucial role of Th17 in the pathogenesis of EAE and as auto-aggressive cells in MS [22–24].

Although we did not observe a similar correlation between CCR6 expression and mean PM10 exposure in 19 healthy controls, we acknowledge that a larger number of healthy individuals would need to be tested before drawing firm conclusions on a possible MS-specific effect of PM10.

Our work suggests that PM can boost Th17 polarization by a DC-dependent IL1 beta, IL6, and IL23 release. To this regard, IL17 was also significantly increased in lung or intestine of rats after experimental PM inhalation or ingestion, respectively [25, 26]. PM10 is known to contain aryl hydrocarbon receptor ligands, and their interaction with the aryl hydrocarbon receptor, which has been shown to play a role in Th17 differentiation and IL17 secretion [27], could represent a possible mechanism of its activity on the immune response.

The present results also imply a crucial role of DC in inflammatory response to PM10. Of note, previous studies similarly found that in vitro urban PM enhanced DC maturation and IL6 production [26, 28]. Importantly, diesel dust, which is over 90% elemental carbon, did not induce their release, thus suggesting that the cytokine-inducing activity of PM10 could be ascribed to different chemical composition of PM compared to diesel dust, including a higher content of extractable organic carbon and inorganic constituents such as sulfates, nitrates, and metals, as well as microbial component. Interestingly enough, in a previous study, pre-treatment of urban air particles with polimixin B and heat shock inactivated its pro-inflammatory effect, thus pointing to a major role of microbial contaminants in proinflammatory effect of PM10 [26].

**Fig. 3:**
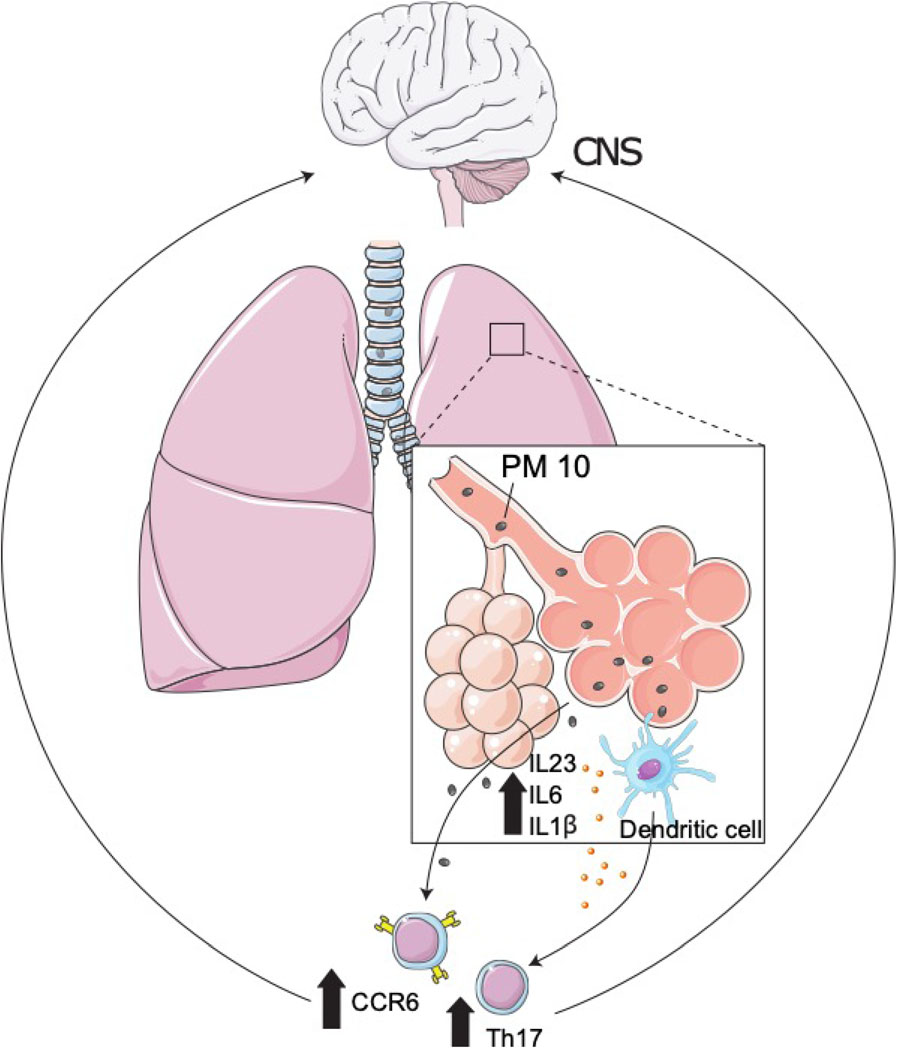
Respiratory exposure to PM10 may lead to increase disease activity in MS patients by inducing a direct upregulation of adhesion molecules and chemokine receptors on circulating lymphocytes and by promoting lung-resident dendritic cell-mediated release of IL1 beta, IL6, and IL23 and subsequent enhanced generation of Th17-producing T cells

Altogether, these observations led us to hypothesize that lung resident DC may uptake PM10 in the lower respiratory tract and, after migration to bronchial associated lymphoid tissue, induce a proinflammatory response with generation of Th17 cells and enhance their migratory properties, ultimately leading to clinical exacerbation of the disease (Fig. 3). This observation parallels previous evidence in the experimental autoimmune encephalomyelitis, the animal model of MS, of a primary role of the lung in licensing auto-reactive lymphocytes to enter CNS by boosting their migratory properties [29].

## Conclusions

The study shows a pro-inflammatory role of PM in MS through upregulation of the expression of CCR6 on circulating CD4+ T cells and induction in innate immune cells of the production of Th17 polarizing cytokines. Evidence is accumulating of a broader effect of airborne pollution on the incidence and progression of inflammatory and infectious disease, including an increased frequency and higher mortality of COVID-19 cases following increased exposure to air pollutants [30, 31]. Larger prospective studies are needed to confirm the effect of PM10 on Th17 cell polarization in MS patients, as well as to confirm the effect of PM10 on migratory properties of circulating lymphocyte and loss of BBB integrity. Future studies should also aim at understanding whether the pro-inflammatory effect of PM10 is MS specific or can also be observed across other autoimmune diseases or in physiological conditions. Also, it is not known whether the observed effect of PM10 depends mainly on its physical properties or on chemical properties of one or more of its components. Finally, molecular pathways underlying the cytokine-inducing activity of PM10 on DC are still largely unknown and will need to be elucidated.

## Data Availability

Anonymized data from this study will be shared by request from any qualified investigator.

## Abbreviations

CCR6: C-C chemokine receptors 6
mdDC: Monocyte-derived dendritic cells
MS: Multiple sclerosis
PBMC: Blood mononuclear cells
PM10: Particulate matter 10
Th17: T helper 17 cells
